# Role of Vitamin D in Colorectal Cancer: A Holistic Approach and Review of the Clinical Utility

**DOI:** 10.7759/cureus.10734

**Published:** 2020-09-30

**Authors:** Moiz Javed, Aldanah Althwanay, Farah Ahsan, Federico Oliveri, Harshit K Goud, Zainab Mehkari, Lubna Mohammed, Ian H Rutkofsky

**Affiliations:** 1 Internal Medicine, California Institute of Behavioral Neurosciences & Psychology, Fairfield, USA; 2 Cardiology, California Institute of Behavioral Neurosciences & Psychology, Fairfield, USA; 3 Medicine, California Institute of Behavioral Neurosciences & Psychology, Fairfield, USA; 4 Internal Medicine, California Institute of Behavioral Neuroscience & Psychology, Fairfield, USA; 5 Psychiatry, California Institute of Behavioral Neurosciences & Psychology, Fairfield, USA

**Keywords:** colon cancer, vitamin d, 1α, 25-dihydroxyvitamin d3

## Abstract

Vitamin D is well known for its effects on the homeostasis of calcium and phosphorus. Lately, considerable research has brought the extra-skeletal role of vitamin D under the spotlight, including its anti-cancer activity. Colorectal cancer (CRC) is the most extensively studied neoplasia that has been observed to be affected by vitamin D; the list includes breast, prostate, and ovarian cancer. This review aims to shine a light on the influence of vitamin D over CRC and to further understand its ability to be used as a potential economical treatment for CRC patients. For this review, PubMed was used as the main database for the literature search. Studies on the role of vitamin D on CRC within 10 years and all of the study types were included. Post the extensive research over PubMed, it was noted that vitamin D, through its effect on multiple pathways, especially Wnt/β-catenin, apoptosis, and inflammation, hinders the progression of CRC carcinogenesis. High levels of this steroid hormone can delay the progression and may provide a cost-effective way of treating CRC patients. Further research and additional human trials are still due to bring about more knowledge on this topic. In conclusion, high serum levels of vitamin D are associated with a lower risk of incidence and progression of CRC.

## Introduction and background

Colorectal cancer (CRC) is the third most common and the second most lethal cancer worldwide. In 2018, 1.8 million new cases and 881,000 deaths were reported for CRC, which accounted for almost 10% of new cancer cases and deaths worldwide [1]. It is projected that there will be nearly 2.5 million new cases by the year 2035 [2]. The initial genetic changes begin in an early adenoma that is initially transformed from the normal colorectal epithelium. Multiple pathways like the chromosomal instability, microsatellite instability, and CpG island methylator phenotype pathways are responsible for the adenoma’s conversion to carcinoma [3]. MLH1, MSH2, MSH6, PMS2, BMPR1A, MSAD4, POLE, NTHL1, MUTYH, POLD1 and adenomatous polyposis coli (APC) are some well-researched germ-line mutations recognized to increase the susceptibility of CRC [4]. So far there hasn’t been a therapy designed that could treat each of the CRCs as one since they all have different driving mutations. Surgery remains to be the leading treatment early on in the disease course but as seen in 25% of the diagnoses when cancer has metastasized, surgery is no longer curative [5]. In patients with stage III CRC, adjuvant chemotherapy with cytotoxic agents plays a critical role as the standard of care [6]. Recent case-controlled studies have displayed an inverse correlation between serum levels of vitamin D and the incidence of human CRC and multiple studies proposed lower incidence of colon cancer, polyp recurrence, and overall survival for patients with colon cancer with higher vitamin D3 levels [7].

Vitamin D is known for its role in regulating bone metabolism primarily through calcium absorption from the intestines and bone remodeling [8]. Endogenous exposure to ultraviolet B radiation stands as the major source of vitamin D for most of the people, which then converts 7-dehydrocholesterol in the skin to vitamin D, later hydroxylated to 25-hydroxyvitamin D, i.e. 25(OH)D, a secosteroid hormone. 25(OH)D is converted to 1,25-dihydroxyvitamin D, i.e. 1,25(OH)2D, the most active metabolite of vitamin D by enzyme 1-α-hydroxylase. Vitamin D is also noted to engage in a variety of physiological pathways including regulation of cell cycle, cellular proliferation, angiogenesis, apoptosis, and molecular cell signaling. This validates its participation in tumorigenic activity [9]. 1,25D3 binds to a specific vitamin D receptor (VDR), which is a member of the nuclear receptor superfamily. VDR then binds to retinoid X receptor (RXR), and the VDR-RXR heterodimers bind to a vitamin D response element (VDRE), which then controls the activation or repression of gene expression [7]. Higher serum levels of vitamin D have been shown to be protective against metastasis in patients with stage IV melanoma. Breast, ovarian, pancreatic, and prostate cancer patients have also benefited from the chemopreventive activities of vitamin D [7]. The literature has supported the relationship in various studies. The overexpression of VDR has been found in CRC and was linked to the phosphatidylinositol 3-kinase (PI3K)-AKT pathway and KRAS mutations [10]. VDR polymorphisms, which include Taql, Bsml, Tru91, have also shown to be possible risk factors for CRC [4]. In this article, our aim is to review the literature concerning vitamin D and CRC mortality, present-day guidelines in regard to vitamin D supplementation, and to address the gaps in the research.

## Review

Method

The main databases used for searching relevant publications were PubMed and Google Scholar. By using the keywords colon cancer, colorectal cancer, vitamin D, 1α,25-dihydroxyvitamin D3, and calcitriol, the search was made with no geographical restriction, for articles on vitamin D and its role in colon cancer. Medical Subject Headings (MeSH) keywords colonic neoplasms and vitamin D was also used for the search. Results yielded were 54,081 peer-reviewed published articles listed for colon cancer, 40,175 peer-reviewed published articles listed for vitamin D, 850 peer-reviewed published articles listed for 1α,25-dihydroxyvitamin D3, 322 peer-reviewed published articles listed for combined keywords vitamin D and colon cancer, 607 peer-reviewed published articles listed for combined keywords vitamin D and colorectal cancer, and 154 peer-reviewed published articles listed for combined keywords calcitriol and colorectal cancer. The search results that were yielded are summarized in Table 1. No restriction of study type, including systemic reviews, investigation of clinical trials, and meta-analysis, was applied in the review. No selection was based on age, gender, and ethnicity. Studies older than 10 years from July 2020 were excluded.

**Table 1:** Search results for the keywords and combinations from PubMed

Keyword	Database	Number of results
Colon cancer	PubMed	54,081
Vitamin D	PubMed	40,175
1α,25-dihydroxyvitamin D3	PubMed	850
Vitamin D and colon cancer	PubMed	322
Vitamin D and colorectal cancer	PubMed	607
Calcitriol and colorectal cancer	PubMed	154

Discussion

Proposed Pathways Involving Vitamin D and CRC

Several potential pathways have been suggested in the involvement of vitamin D in CRC, including its role in apoptosis, initiation of differentiation in colonic epithelium, and suppression of angiogenesis. Mutation in the APC tumor suppressor gene is the primary genetic change in the majority of colorectal adenomas, which is a primary step in the development of CRC. To a smaller extent, AXIN2 and CTNNB1/β-catenin genes are also involved. Additionally, activating mutation in the KRAS and BRAF, and an inactivating mutation in the transforming growth factor (TGF)-β pathway plays a role in the malignancy of adenoma cells [11].

**Figure 1:**
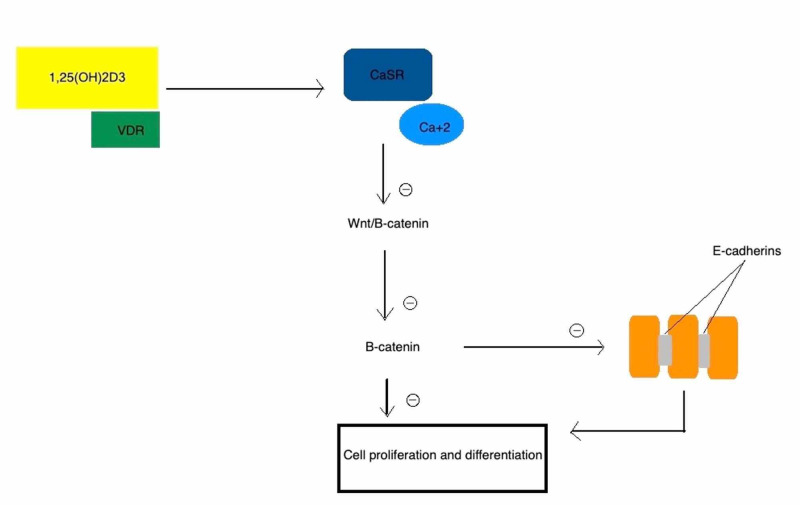
Vitamin D and its effects on the Wnt/β-catenin pathway and E-cadherins VDR: vitamin D receptor

According to recent studies, the up-regulation of expression of the calcium-sensing receptor (CaSR), an important player in cell proliferation, has been proposed in the list of antineoplastic effects of vitamin D [12]. CaSR interacts with the cell cycle of colonocytes via inhibition of the β-catenin/T-cell factor (TCF) transcription complex, promoting E-cadherin activation and lowering the concentration of 25-hydroxyvitamin D 24-hydroxylase (CYP24A1) [13]. A study conducted by Aggarwal et al., in which in vivo mice were fed with a high vitamin D diet, showed a notably higher amount of CaSR expression, decreased quantity of colonic proliferating cells, and unregulated apoptosis [14]. Wnt signaling pathway is an established contributor to the progression of CRC [15]. The accelerated growth of the tumor and malignancy were contributed to the hyperactive Wnt/β-catenin pathway after the loss of VDR. As compared to healthy colon cells, there was a notable increase in the colonic Wnt and β-catenin stained cells in colorectal and colitis patients, with an increase of 4.5 folds and 2.5 folds, respectively [16]. 1,25D suppresses β-catenin transcriptional activity. Independently without the involvement of the VDR DNA binding site, 1,25D suppresses the transcriptional activity of β-catenin and expression of the β-catenin target gene DDK-4. In the presence of APC, a tumor suppressor, which represses the Wnt pathway, the activity of 1,25D against β-catenin is enhanced [7]. Larriba et al. supported the direct relation between nuclear β-catenin levels and VDR functions in controlling the activity of Wnt/β-catenin signaling in colon cancer. The effects were shown both in vivoand vitro with the help of Apcmin/+Vdr-/- mice and cultured human colon cancer cells in which shRNA was used to knock down the expression of VDR. The study reported induction of the expression of E-cadherin and antagonization of the Wnt/β-catenin pathway to enhance epithelial differentiation and inhibition of proliferation of human colon cancer cells by 1,25(OH)2D3 [17]. Figure 1 illustrates these effects. E-cadherin is an integral protein involved in epithelial cell adhesion. Its loss results in the conversion of a more invasive phenotype of an epithelial cell from its normal phenotype. E-cadherin for that reason is considered as a proto-oncogene [18]. Furthermore, secreted frizzled-related protein 2 (SFRP2) is an inhibitor of Wnt and its prompted CpGs have been documented to methylate at a high rate in CRCs [19]. Vitamin D decreases the promoter methylation of SFRP2, which then antagonizes Wnt, which could help in the ability of vitamin D to work with neoadjuvant treatments and improve their response in patients with CRC [20].

Vitamin D also affects the process of apoptosis in CRC cells. Apoptosis is programmed cell death, a process through which all the multicellular organisms go through [21]. These cells go through characteristic changes in their morphology as a part of the process of apoptosis, these include blebbing, fragmentation of the nucleus, shrinkage of the cell, condensation of the chromatin, and fragmentation of the chromosomal DNA [22]. 1,25(OH)2D3 in colorectal adenomas and carcinoma causes the downregulation of BAG1, an anti-apoptotic protein in the nucleus, and the up-regulation of a proapoptotic protein BAK1. It also causes an increase in the expression of G0S2, which is a mitochondrial protein that interacts with Bcl-2 to induce apoptosis in CRC cells by preventing the formation of anti-apoptotic heterodimers between Bcl-2 and BAX [11]. Colonic epithelial VDR inhibits apoptosis in the epithelium by downregulating p53 upregulated modulator of apoptosis (PUMA), an important proapoptotic regulator that protects the mucosal barrier of the colon and reduces inflammation [23]. Moreover, various inflammatory pathways that take part in the progression of CRC such as the nuclear factor (NF)-κB and cyclooxygenase pathways, and many cytokines, such as tumor necrosis factor (TNF)-α, interleukin (IL)-1β, IL-6, IL-8, IL-17, and TGF-β1, have shown to be affected by vitamin D. CRC patients have higher serum levels of these cytokines. A randomized, double-blind, placebo-controlled, 2 x 2 factorial trial by van Harten-Gerritsen et al. studied patients with colorectal adenoma, which were given 800 international units of vitamin D3 and/or 2 g calcium versus placebo for over six months. Although not statistically significant, post-six-month baseline measurements of plasma inflammatory markers and serum vitamin D displayed high levels of vitamin D with decreased levels of CRP, TNF-α, IL-1β, IL-6, and IL-8 in the group that received vitamin D. The group that received the calcium and vitamin D had decreased levels of IL-1β, IL-6, and IL-8 [24].

Serum Levels of Vitamin D and Risk of CRC

The association between mortality in CRC patients and lower vitamin D levels resulting from weak ultraviolet-B radiations at high altitudes was first put forward by Garland and Garland in 1980 [25]. Since then, many studies have supported this relationship. Most of the patients who were newly diagnosed with CRC were found to have either deficient or insufficient levels of 25(OH)D [26]. Studies have also suggested restoring the level of 25(OH)D to normal levels of 30-80 ng/mL in CRC patients who are deficient and regularly monitoring its level [27]. In Intergroup Trial N9741, Ng et al. concluded that stage IV CRC patients who were receiving chemotherapy as first-line treatment, particularly female and Black patients, had a high prevalence of vitamin D deficiency. With levels ≥30 ng/mL of 25(OH)D levels considered as sufficient, 25(OH)D levels lower than 20 ng/mL were defined as vitamin D deficiency, and 20-29 ng/mL were regarded as insufficiency. In the study, 82% of the study population were vitamin D insufficient and 50% were deficient in vitamin D [28].

A nested case-control study by Chandler et al. included 274 female CRC cases and 274 female controls and followed the study population for 16.3 years to find out that those with 25(OH)D levels above 29 ng/mL were seen to have the highest reduction in the incidence and mortality with CRC [29]. The serum level of vitamin D and its effect on CRC-related mortality have also been highlighted by the dose-response analysis involving 17,700 participants from 18 studies by Wu et al. The study found a 12% lower risk of colorectal-specific mortality and a 7% reduction in the risk of all-cause mortality with an increase of every 20 nmol/L of 25(OH)D [30]. Moreover, data from 17 cohorts, including 5706 CRC patients and 7107 control participants, were used in a study by McCullough et al. and showed that the most favorable concentration of 25(OH)D for risk reduction of CRC was 75-100 nmol/L, which is higher than the current Institute of Medicine (IOM) recommendations. The study also highlighted that the higher levels of circulating 25(OH)D had a statistically significant effect on women and a non-statistically significant effect on men for lowering the risk of CRC [31]. The reduced risk of CRC with vitamin D serum levels in male and female patients from diverse ethnic groups such as African American, Latino, White, Native Hawaiian, and Japanese has also been observed [32]. To further explore the effects in different races, another meta-analysis with CRC patients in Asian countries was conducted by Zhang et al. that showed a statistically significant reduction in the risk of CRC by 21% for every 16 ng/mL increase in serum vitamin D levels with an odds ratio (OR) of 0.79 (95% CI 0.64 to 0.97) [33]. However, low-dose vitamin D supplementation of 400 IU per day did not benefit in the reduction of CRC risk as shown by Women’s Health Initiative (WHI), a randomized placebo-controlled trial [34].

Vitamin D as a Potential Treatment in CRC

The overall survival of patients with CRC is directly related to higher circulating levels of 25(OH)D. With this reduction in the all-cause and CRC-specific mortality, 25(OH)D can be an excellent protective agent that could positively affect the prognosis in these patients [30]. 25(OH)D-derived analogs have been developed to provide more potent antineoplastic effects and counter the unwanted calcemic side effects that have been a problem with using 25(OH)D alone as a treatment for cancer [35]. PRI-2191, a vitamin D analog, increases the expression of E-cadherin and arrests the cells in the G0/G1 cell cycle phase. It is noted to work alongside 5-fluorouracil (5-FU), the oldest drug used to treat CRC, in enhancing and extending its anticancer effects inin vivo mice. PRI-2191 can be used in the colon cancer treatment regimen to increase the effects of 5-FU [36]. SUNSHINE, a double-blinded, multicenter, phase II randomized clinical trial with untreated 139 colorectal patients gave one group of study participants 400 IU of vitamin D per day and the other group 4,000 IU vitamin D per day. Both groups also received standard chemotherapy. The results showed a median delay time of 13 months for the worsening of disease progression in those receiving the high-dose vitamin D and 11 months in the low-dose vitamin D group. Furthermore, those in the high-dose group had a 36% less chance of CRC progression or death during the follow-up time of 22.9 months [37]. However, adding 2,000 IU of cholecalciferol per day into the standard chemotherapy regimen of metastatic cancer patients for two years in another study did not produce any advantages in the overall survival or the progression-free survival with a follow-up period of 46 months [38]. With the results from many preclinical and clinical studies, the inverse relationship between vitamin D deficiency and the risk of developing CRC has been advocated. Vitamin D addition can be an economical and secure way of reducing the incidence and improving prognosis in CRC despite the variable support from the existing early studies. More randomized control trials in humans are warranted to solidify the relationship [39].

Table 2 shows the studies used in the review and their relevant findings.

**Table 2:** Studies included in this review with their pertinent findings 25(OH)D: 25-hydroxycholecalciferol; APC: adenomatous polyposis coli; CRC: colorectal cancer; COX2: cyclooxygenase-2; 5-FU: 5-fluorouracil; IL: interleukin; NF-κB: nuclear factor-κB; TNF-α: tumor necrosis factor-α; TGF-β1: transforming growth factor-β1; VDR: vitamin D receptor

Author	Year of publication	Study design/type of study	Topic	Findings
Ng et al. [28]	2011	Intergroup trial N9741	Vitamin D status in patients with stage IV CRC	Stage IV CRC patients had a high prevalence of vitamin D deficiency.
Larriba et al. [17]	2011	Animal model colon cancer APC min/+ mice	VDR deficiency enhances Wnt/β-catenin signaling and tumor burden in colon cancer.	VDR is a key participant of the Wnt/β-catenin pathway in controlling tumor growth in CRC.
Pereira et al. [11]	2012	Review	Vitamin D and colon cancer	Vitamin D has a protective role against CRC especially in the prevention.
Milczarek et al. [36]	2013	In vivo mouse model	Vitamin D analogs enhance the anticancer activity of 5-FU in an in vivo mouse colon cancer model.	With vitamin D analogs PRI-2191 and PRI-2205, the activity of 5-FU was appreciably increased with a decreased growth of the tumor, metastasis, and increased survival in the mice.
Klampfer [7]	2014	Review	Vitamin D and colon cancer	Calcitriol through its interaction with VDR inhibits Wnt signaling pathway and exerts its anticancer effects.
van Harten-Gerritsen et al. [24]	2015	Review	Vitamin D, inflammation, and CRC progression	Vitamin D controls cytokines, TNF-α, IL-1β, IL-6, IL-8, IL-17, and TGF-β1 and COX2 and NF-κB pathways in CRC.
Ng et al. [37]	2019	Randomized clinical trial	Effect of high-dose vs standard-dose vitamin D3 supplementation on progression-free survival among patients with advanced or metastatic colorectal cancer	Increased progression-free survival in CRC patients from 11 to 13 months when high-dose vitamin D used with chemotherapy vs chemotherapy alone
McCullough et al. [31]	2019	International pooling project of 17 cohorts	Circulating vitamin D and CRC risk	75-100 nmol/L of 25(OH)D is optimal for reducing CRC risk in women and men.

Limitations

Most of the studies included are animal studies done on mice. Some of the human studies had a limited number of participants involved. There is a need for further extensive human trials to get more insight into the effects of vitamin D on colon cancer for it to be included in the treatment plan for the prevention of high-risk patients and those with diagnosed colon cancer.

## Conclusions

1,25D3 after binding to its receptor, VDR, exerts its anti-neoplastic effects by changing the expression of multiple genes. Vitamin D has a pro-apoptotic and anti-inflammatory effect, in addition to its inhibition of the Wnt/β-catenin, to decrease the growth and differentiation of colon epithelial cells. Studies suggest the increase in the incidence of CRC with low serum levels of vitamin D. Higher levels of vitamin D have been suggested to prevent CRC and to decrease the mortality in CRC patients; meanwhile, the addition of low-level vitamin D did not show significant results. The ability of vitamin D analogs to work alongside the already established CRC treatments, like 5-FU, would provide an economical additional treatment for such patients. However, additional large-scale human trials are warranted to further investigate this relationship to clearly devise a more systematic treatment plan that includes vitamin D.
